# Hypnotics Use Is Associated with Elevated Incident Atrial Fibrillation: A Propensity-Score Matched Analysis of Cohort Study

**DOI:** 10.3390/jpm12101645

**Published:** 2022-10-04

**Authors:** Xiang Hu, Gwo-Ping Jong, Liang Wang, Mei-Chen Lin, Shao-Qing Gong, Xue-Hong Zhang, Jiun-Jie Lin, Esther Adeniran, Yan-Long Liu, Hung-Yi Chen, Bo Yang

**Affiliations:** 1 Department of Endocrine and Metabolic Diseases, Key Laboratory of Clinical Laboratory Diagnosis and Translational Research of Zhejiang Province, The First Affiliated Hospital of Wenzhou Medical University, Wenzhou 325000, China; 2 School of Public Health and Management, Wenzhou Medical University, Wenzhou 325035, China; 3 Division of Cardiology, Department of Medicine, Chung Shan Medical University Hospital, Chung Shan Medical University, Taichung 402, Taiwan; 4 Department of Public Health, Robbins College of Health and Human Sciences, Baylor University, Waco, TX 76798, USA; 5 Management Office for Health Data, China Medical University Hospital, Taichung 404, Taiwan; 6 College of Medicine, China Medical University, Taichung 404, Taiwan; 7 School of Public Policy and Administration, Xi’an Jiaotong University, Xi’an 710049, China; 8 Department of Nutrition, Harvard T.H. Chan School of Public Health, Boston, MA 02115, USA; 9 Department of Pharmacy, Feng-Yuan Hospital, Ministry of Health Welfare, Taichung 420, Taiwan; 10 Department of Biostatistics and Epidemiology, College of Public Health, East Tennessee State University, Johnson City, TN 37614, USA; 11 Department of Pharmacy, China Medical University, Taichung 404, Taiwan; 12 Department of Pharmacy, China Medical University Beigang Hospital, Yunlin 651, Taiwan

**Keywords:** atrial fibrillation, hypnotics, cohort study, benzodiazepines

## Abstract

We aimed to investigate the association between either or both of benzodiazepines (BZDs) and non-BZDs and the incidence of atrial fibrillation (AF) in the Taiwan National Health Insurance Database. The participants with at least two prescriptions of BZDs and/or non-BZDs were identified as hypnotics users, whereas those without any prescription of hypnotics were non-hypnotics users. The hypnotics and non-hypnotics cohorts were 1:1 matched on their propensity scores. A total of 109,704 AF-free individuals were included; 610 AF cases occurred in the 54,852 hypnotics users and 166 in the 54,852 non-hypnotics users during the 602,470 person-years of follow-up, with a higher risk of new-onset AF in the users than the non-users (hazard ratio (HR): 3.61, 95% confidence interval [CI]: 3.04–4.28). The users at the highest tertiles of the estimated defined daily doses per one year (DDD) had a greater risk for AF than the non-users, with the risk increasing by 7.13-fold (95% CI: 5.86–8.67) for >0.74-DDD BZDs, 10.68-fold (95% CI: 6.13–18.62) for >4.72-DDD non-BZDs, and 3.26-fold (95% CI: 2.38–4.47) for > 1.65-DDD combinations of BZDs with non-BZDs, respectively. In conclusion, hypnotics use was associated with elevated incidence of AF in the Taiwanese population, which highlighted that the high-dose usage of hypnotics needs more caution in clinical cardiological practice.

## 1. Introduction

Atrial fibrillation (AF) is the most commonly sustained rhythm-disorder encountered in clinical practice, which is currently highlighted by a high prevalence and serious clinical consequences, such as hemodynamic impairment and ischemic stroke. Globally, the prevalence of AF/atrial flutter increased from 19.1 million in 1990 to 37.6 million in 2017, demonstrating the emergence of AF as a global epidemic [1]. According to the data from the National Health Insurance Research Database (NHIRD), the prevalence rates of AF are estimated to increase from 1.51% to 4.0% in Taiwan from 2020 to 2050 [2]. 

Hypnotic drugs are one of the most commonly prescribed classes of psychotropic drugs [3], consisting of traditional benzodiazepines (BZDs) and new generational nonbenzodiazepines (non-BZDs), colloquially as Z-drugs [4]. The traditional BZDs including diazepam, lorazepam, triazolam, oxazepam, and midazolam, are widely used in the treatment of psychiatric disorders, while prescriptions of the non-BZDs, including zaleplon, zopiclone, and zolpidem, have been greatly promoted over BZDs, because of their superior safety as a short-acting hypnotic drug. In Taiwan, the use of hypnotics seems to increase with age, with a relatively high proportion of the prescriptions of zolpidem, a type of non-BZD [5]. 

The evidence from data at cellular and animal levels have well documented a biological link between the use of hypnotics and decreased risks of cardiovascular disease (CVD). In hypoxia/reoxygenation-mediated H9C2 cells, midazolam was found to retard the I/R-induced cardiomyocyte apoptosis by inhibiting the JNK/p38 MAPK-signaling pathway [6]. The endothelium-independent vasorelaxation in response to most of the BDZs and two non-BDZs (zaleplon and zolpidem) was determined by myograph methods, using isolated thoracic aortas in the Wistar rat [7]; however, the direct vasodilatory effects of these drugs may be involved in the mechanisms underlying their adverse effects, such as a potential decrease in blood pressure. The above-mentioned laboratory studies may have underlain the pharmacological basis of the potential association of hypnotic drugs with cardiovascular risk. Nevertheless, the epidemiological studies have not reached an agreement in clinical findings, with no change or an increase in all-cause mortality for patients with prescribed use of BZDs [8,9,10,11,12]. Moreover, the effect for cardiovascular mortality perhaps differed by subtype of hypnotic agents [8], and extended to the patients even with low-dose use. There is a current gap in the knowledge of the possible relationship between the prescribed use of hypnotics and the risk of AF. So far, no clear conclusion can be drawn from the limited existing population-based cohorts to support a specific dose-response effect of BZDs or non-BZDs that could explain the increase in the AF risk. 

The present population-based cohort aimed to investigate the association between the prescribed use of hypnotics, including either or both of BZDs and non-BZDs and the subsequent risk of AF, using the data from the NHIRD and mitigating the potential impacts of the intrinsic confounders through specific analytic strategies, such as propensity score matching.

## 2. Materials and Methods

### 2.1. Data Source

The NHIRD was established in Taiwan in 1995, and its details were published previously [13,14]. Briefly, the contents of the database come from the single-payer health insurance program covering more than 99% of 23 million persons in Taiwan, which provides information on the comprehensive medical services in outpatient and inpatient care, physical therapy, preventive care, and prescriptions. The National Health Research Institute has been responsible for the administration of the NHIRD and released the claim-file database, representative of one million individuals (Longitudinal Health Insurance Database 2000), randomly selected from all of the insured registered in the NHIRD from 1996 to 2009. The National Health Research Institute reported no statistical differences in the distributions of age, gender, or health care expenditures between the subset of the NHIRD and all of the enrollees. 

### 2.2. Study Design

The retrospective cohort analyses were conducted by using the Longitudinal Health Insurance Database 2000, which randomly selected one million study subjects from the NHIRD. We were able to use a scrambled, anonymous identification number for each of the insured to link the individual’s medical records, including the registry of medical services, medications prescribed, inpatient orders, and preventive care. The available socio-demographic information for the study subjects included gender, birth date, and residential area. All of the diagnoses recorded in the database were coded by the International Classification of Disease, Ninth Revision, Clinical Modification (ICD-10-CM). This study approval was granted by the Research Ethics Committee of China Medical University and Hospital in Taiwan (CMUH-104-REC2-115(CR3)). 

### 2.3. Study Population

The eligible participants were those aged 20 years and older, permanently registered with a practice contributing data to the NHIRD, and with at least 12 months to be followed up for standard records. The selection flow of the study participants is presented in the Figure S1 (Supplementary Materials). In total, 88,095 participants with at least two prescriptions of hypnotics between 1 January 2004 and 31 December 2012 were eligible for the prescribed-hypnotics cohort, including either of BZDs and non-BZDs, or both, whereas those without any use history of the hypnotics prescriptions were non-users and defined as the comparison cohort. The patients’ initial date of the prescribed use of hypnotics was set as the index date.

We excluded individuals if they received the prescription for BZDs and/or non-BZDs before 2004, or had a history of AF (ICD-10-CM codes 427·31) diagnosed before the index date or missing information. The exposure propensity scores were derived from the predicted probability of hypotonic treatment, using a logistic regression model that contained most of the patients’ characteristics (the index date, age, gender, regions, comorbidities, and concomitant medications) without additional variable selections. The hypnotics and the control cohorts were 1:1 matched on their propensity scores, using a nearest neighbor matching algorithm without replacement.

### 2.4. Ascertainment of Hypnotics Use

We ascertained the receipt of hypnotic drugs from the electronic prescribing records in the NHIRD. The use was initially quantified in terms of the defined daily doses from each patient’s initial date of hypnotic prescription to the end of their follow-up period. The hypnotics users were those who received at least two prescriptions for a given study drug, which helped to minimize the hypnotics misclassification among the patients who received, but did not fill, the prescription or take the drug. We also calculated the duration of each prescription by dividing the number of tablets prescribed by the number to be taken each day. If no gaps of more than 90 days existed between the end of one prescription and the start of another, the patients were classified as exposed to the use of hypnotics. If gaps of more than 90 days occurred, the patients counted as exposed for the first 90 days, and then unexposed for the remaining period. For the subclass analyses, the hypnotics were grouped according to three main classes in the Taiwan National Formulary: BZD alone; non-BZD alone; and their combinations without priorities, and the prescriptions for different drugs were classified on the same date as the combined prescriptions. All of the names of the study hypnotics with ATC codes are listed in Table S1 (Supplementary Materials). 

We calculated the daily dose of each prescription by multiplying the number of tablets to be taken each day by the dose of each tablet, and converted this to a defined daily dose (DDD) to enable the comparison of doses between the hypnotics classes, using values assigned by the WHO Collaborating Center for Drug Statistics Methodology [15]. The DDD was estimated by dividing the cumulative defined daily dose during the follow-up period of study by the duration of each prescription to assume an average maintenance dose per day for a drug used for its major indication in one adult, permitting the combination of the usage data across the different drugs used for the same indication. 

### 2.5. Ascertainment of Outcome Measurements

The analytic outcomes were the incidence of AF. We identified the patients with new-onset AF if they were recorded on their general practice record using the ICD-10 diagnostic code (ICD-10-CM: 427·31). For the analysis of the AF outcome, we considered only the first event and excluded patients with a previous diagnosis of AF recorded at baseline. 

The hypnotics and non-hypnotics cohorts were followed up from the index date to December 31, 2013, the date of AF onset, when withdrawn from the insurance, or death, depending on which date came first. The mean of the follow-up years for the hypnotics and non-hypnotics cohorts were 5.81 and 5.16, respectively.

### 2.6. Ascertainment of Analytic Covariates

We calculated the absolute differences to assess covariate balance before and after propensity scores matching. In addition to age, gender, and living regions, analytical covariates included comorbidity-related variables and medication-related variables. The clinical comorbidity-related covariates included: overweight/obesity (ICD-10-CM: 278); diabetes (250); and CVD including hypertensive heart disease (402), ischemic heart disease (410–414), pericardium disease (420–424), cardiomyopathy (425), cardiac dysrhythmias (426–427), heart failure (428), complications of heart disease (429), cerebrovascular disease (430–438), chronic obstructive pulmonary disease (COPD) (491, 492, 496), anxiety (300), and sleep disorder (307, 327, 780.5). The medication-related covariates included historical record of usage of concomitant drugs, such as statins (ATC code: C10AA01-C10AA08), glucocorticoids (H02AB, R03BA), non-steroidal anti-inflammatory drugs (D11AX18, M01A, M01B), antithrombotic drugs (B01AC04, B01AC24, B01AC06), anticoagulants (B01AA03, B01AF01, B01AE07), antidiabetic agents (A10A, A10B, A10X), and anti-hypertensive drugs (C09AA, C09CA, C03, C08CA, C08D, C07A). 

### 2.7. Statistical Analysis

The normality of the data distribution was accessed by the one-sample Kolmogorov–Smirnov test. The categorical data were shown by numbers and percentages, while the continuous data were shown either by mean ± standard deviation (SD) for normal-distribution data, or by a median with an interquartile range 25–75% for the skewed data, respectively. The difference in the baseline categorical and continuous variables between the two cohorts was tested by chi-square test and *t*-test, respectively. The risk of developing AF in the users compared to the non-users was estimated by using a multivariate-adjusted Cox proportional regression model treating the prescribed use of hypnotics as a time-varying exposure to allow for patients starting and stopping and also changing between treatments throughout the follow-up, presented by multivariate-adjusted hazard ratios (HRs) with 95% confidence intervals (CIs). Additional analyses were carried out for time-varying exposures of the prescribed daily dose, categorized as the tertiles of the estimated DDD for each hypnotics class, and we performed tests for the trend within each drug class by using the dose as a continuous variable. The cumulative incidences of AF were demonstrated by Kaplan–Meier analyses, and the difference between the two cohorts was compared by using a log-rank test. 

The consistency of the overall findings was assessed in the potential subgroups pre-defined by the unbalanced baseline variables between the users and non-users before propensity scores matching. Furthermore, interaction tests were conducted to test whether multivariable-adjusted HRs statistically differed between the strata analyzed, by simultaneously including each strata factor, the use of hypnotic agents, and the respective interaction terms (the strata factor multiplied by hypnotic use) in the Cox regression model. All of the significant criteria were set at a two-tailed *p* value < 0.05, and the statistical analyses were performed using SAS statistical software, version 9·4 (SAS Institute Inc., Cary, NC, USA).

## 3. Results

The demographic characteristics, clinical comorbidities, and the usage of concomitant medications between the participants with and without prescribed use of hypnotics are shown in Table 1. After using the propensity score-matched analysis with a 1:1 ratio, 54,852 hypnotics users and 54,852 non-hypnotics users were eligible for the present analysis. Among the 109,804 participants, 51.2% were males, and the mean age was 43.44 years. No statistical difference was found in the gender, age, living regions, clinical comorbidities, and concomitant medications between the users and non-users.

**Figure 1:**
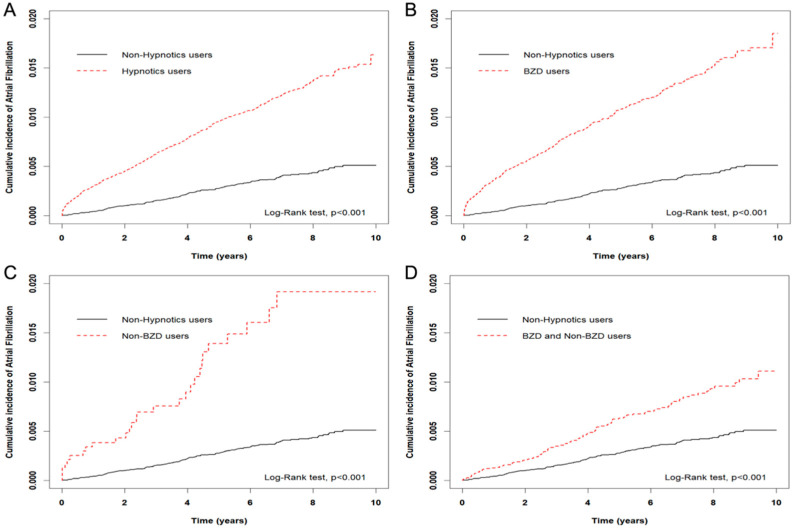
Comparisons of Kaplan–Meier estimates for the cumulative incidence rate of atrial fibrillation between hypnotic and non-hypnotic cohorts. Using the log-rank test, the cumulative incidence rates of atrial fibrillation (AF) were higher in participants with prescribed use of either or both of benzodiazepines (BZDs) and non-BZDs (**A**), isolated BZDs (**B**), isolated non-BZDs (**C**), and their combinations without priorities (**D**) than those with no prescription of hypnotics (*p* <0.001, respectively).

Over the 602,470 person-years of follow-up duration, 610 AF events occurred in 54,852 hypnotics users and 166 in 54,852 non-hypnotics users (Table 2), with a higher incidence rate of AF in the users compared with the non-users (19.11 versus 5.86 per 10,000 person-years, *p* < 0.001). There was a significantly higher cumulative incidence in the participants with the use of BZDs alone (1.22%), non-BZDs alone (1.50%), or their combinations (0.81%) compared with those without any use of hypnotics (0.30%), respectively (Figure 1). 

**Figure 2:**
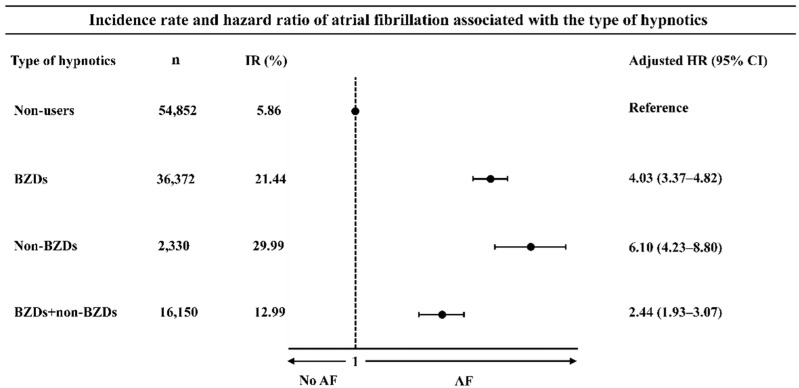
The subsequent risk for atrial fibrillation associated with use of either or both of benzodiazepines and non-benzodiazepines. Hazard ratios (HR) for the incident atrial fibrillation (AF) comparing the users with either or both of benzodiazepines (BZDs) and non-BZDs with non-hypnotics users were estimated by the Cox proportional model with multiple adjustments for socio-demographic factors, clinical comorbidities, and concomitant medications at baseline. The multivariate-adjusted HRs for the prescribed use of BZDs alone, non-BZDs alone, or their combinations without priorities were represented by black diamonds, and their confidence intervals (CIs) were represented by error bars.

The participants with the use of isolated BZDs, isolated non-BZDs, or their combinations without priorities had a 3.61-fold higher risk for AF than the non-users (HR: 3.61, 95%CI: 3.04–4.28). The multivariate-adjusted HRs were 4.03 (95%CI: 3.37–4.82) for the isolated BZDs, 6.10 (95%CI: 4.23–8.80) for the isolated non-BZDs, and 2.44 (95%CI: 1.93–3.07) for their combinations, respectively (Figure 2). 

When stratified by gender, age, living regions, clinical comorbidities including CVD, and concomitant medications, the elevated risks of AF in the user cohort compared with the non-user cohort remain significant in most of the subgroups, with no significant interactions between the hypnotics use and the strata analyzed (Table 2). 

The estimated dose–response association between hypnotics use across the tertiles of DDD and the incident AF was shown in Table 3. Compared to the non-users, the risk of AF was significantly elevated with the increasing dosage of hypnotics used, with the risk increasing by 7.13-fold (95% CI: 5.86–8.67) for > 0.74-DDD BZDs alone, 10.68-fold (95% CI: 6.13–18.62) for > 4.72-DDD non-BZDs alone, and 3.26-fold (95% CI: 2.38–4.47) for > 1.65-DDD combinations of BZDs and non-BZDs, respectively.

## 4. Discussion

To the best of our knowledge, the current study was the first population-based cohort that employed the propensity score-matched analysis to evaluate the association between the use of hypnotics and the risk of subsequent AF in the Taiwanese population. Our major findings demonstrated that the participants with the use of either BZDs or non-BZDs, or their combinations were more vulnerable to the onset of AF than those without use, and there was a sharp rise in the risk for AF in the hypnotics use at a higher level of dosage. Such findings fill the current gap that lacks detailed associations for the incidence of AF in populations with long-term prescribed use of hypnotics.

There are several possible reasons for interpreting this observed association between use of study drugs and the increased risk of AF. The patients with a long-term use of hypnotics may have more hypnotics-related adverse effects than non-users, leading to a higher risk of psychiatric disorders, cardiovascular disease, dementia, and so on [16], which may have increased the likelihood of developing AF. Another possible explanation is that the patients who are already at a high risk for AF may have a greater tendency to start using BZDs or non-BZDs than those at no or low risk. For instance, patients with depression or anxiety, which were associated with an increased risk of the incidence of AF [17], frequently suffer from insomnia and thus, are more predisposed to the use of hypnotics. In addition, the hypnotic users have more visits to their physicians than non-users, because of the given higher risks of AF, and this may explain that hypnotic users are subjected to more ECGs and a higher detection rate of AF. However, in the present study, the individuals with and without use of hypnotics were matched by using the propensity scores approach in respect of demographic characteristics, comorbidities, and medications’ usage. Therefore, the possibility might have been excluded that the direction of estimated association would be changed by the unbalanced distributions of prevalent psychosomatic comorbidities between the users and non-users’ cohorts. Further stratified analysis with interaction tests confirmed that there is no interference from these strata variables with the increased risks of AF brought about by the prescribed use of hypnotics. The present findings suggested that the use of hypnotics, independently and directly, would increase the risk of AF when removing their cardiovascular benefits of relieving anxiety and sleeping disorders, as well as the interaction with other medications. 

We considered the possibility that the cardiac effects varied between different types of hypnotic agents and found either BZDs or non-BZDs leads to a greater risk for developing AF than the non-users. Similar with our finding, a modest increase in cardiovascular mortality in the hypnotic users of either BZDs or Z-drugs (a kind of non-BZD) was seen in a European population-based study of women aged more than 50 years [11]. Indeed, the detrimental effect on the cardiac autonomic function may be partially attributable to the BZDs’ potentiation of the GABAergic neurons that directly inhibit the sympathetic and parasympathetic-related nuclei in the brain stem. In addition, a meta-analysis supported that zolpidem exhibited a tendency for a decreased risk of heart disease [8]. However, when the eligible studies with multiple confounders, such as smoking, were included in the meta-analytic multivariable-adjusted model, the favorable effect for the zolpidem may have disappeared. In a rat study with a 12-h/12-h light/dark photoperiod circle, the fluctuation of heart rate would be intensified with a lower dose of zolpidem, but attenuated with a higher dose of zolpidem in the dark period [18]. In support of this idea, we also consistently observed that the patients with a high-dose use of non-BZDs seemed to be more vulnerable to developing AF than those with a low-dose use, suggesting a possibility of cardiac arrhythmia would increase with the high-dose use in clinical practice. Unfortunately, the present study did not intend to examine the mechanisms underlying the differential effects of BZDs and non-BZDs on AF events. Moreover, our data showed that the patients with use of non-BZDs alone seemed to have a higher multivariate-adjusted HR than those with use of BZDs alone. However, the confidence intervals for the two HRs were at least, or in part, overlapped, with no statistical significance in the cumulative incidence rates for AF events between BZDs alone and non-BZDs alone groups (445/36361 vs. 35/2330). In addition, when the BZDs and non-BZDs were combined, the adverse impacts upon AF risk seemed to be slightly decreased compared with either use of BZDs or non-BZDs. A possible explanation was that their combinations were without priorities in the time sequence, which may have at least or in part counteracted the adverse effects of BZDs alone or non-BZDs alone. The pharmacology mechanisms behind the potential impacts of the mixed use of the hypnotics were not fully understand. Well-designed laboratory investigations are further needed to clarify this issue. 

Previous findings from the NHIRD showed that the risk of ischemic heart disease increased in proportion to the hypnotic dose among psoriasis patients [19]. Similarly, the present study observed that, within the range of lower dose, the risk of AF remained stable or increased a little with an increase in the dose of BZDs or non-BZDs. However, the risk of AF was almost tripled when the dose exceeded a certain range (> upper tertile value), suggesting that at least one-third of hypnotics users suffered a much higher risk of AF because of the use of hypnotics with over-dosing. Since the long-term use of hypnotics increased with age [20], there was no doubt that DDD would be higher among the elderly, who were more prone to AF and a worse prognosis [21]. Therefore, the clinical use of hypnotics should be restricted under a certain safety threshold, and special attention should be paid to the elderly population.

The biological mechanism through which hypnotics would lead to an increased risk of AF remained unclear. There were several mechanistic points that may have potentially explain our present findings (Figure S2, Supplementary Materials). Animal research showed that a hypnotic injection could result in adverse effects on the cardiovascular parameters, such as blood pressure and heart rate in rats [18]. Moreover, for rats that were maintained on a 12-h/12-h light/dark photoperiod schedule, the administration of diazepam induced both a hypertensive effect and tachycardia in the light period. The previous findings in rats showed that a low dose of zolpidem exerted a vagotonic effect, but a high dose of zolpidem inversely behaved as a vagolytic agent. It was suggested that the differential cardiac effect might be related to the selective affinity of Z-drugs to the a1 subunit of GABAA [22]. Zolpidem prolonged the cardiac action via the inhibition of the human ether-a-go-go-related gene (hERG) K^+^ channels [23]. In addition, a type of BZD functioned as a typical open-channel inhibitor of cardiac Kv1.5 channels underlying the atrial repolarizing current I(Kur), with rapid onset of block and without frequency dependence of block [24]. BZDs were suggested as regulating the normal KCNQ1 K^+^ channels, as well as the normal and arrhythmia-associated mutant KCNQ1 K^+^ channels [25].

One of the strengths of this study is the use of a large population-based cohort, with more than 100,000 individuals to compare the subsequent AF risk between cohorts of hypnotics users and non-users, which attempted to ensure the temporal causality between the exposure (hypnotics use) and the endpoint (AF events). Further strengths are the recruitment strategy and ascertainment of drug use, based on documented prescriptions rather than self-reported receipt or use, which may minimize the misclassification by excluding patients with only one prescription. Using the DDD to quantify the cumulative use of the study drugs allowed us to combine the effects of different drugs in a way that is not possible by counting prescriptions or pills, thereby increasing the accuracy of the estimations of drug use, as well as ensuring our results are generalizable to all of those who receive hypnotic drugs in health care. Furthermore, we applied the propensity score-matching approach to minimize the possibility of indication bias that may have caused confounding and threaten the validity of the inherent representativeness, which may have resulted in a more robust estimation. 

The main limitation of the present study is the confounding factors. First, the present study was entirely dependent on the administrative claims’ data from NHIRD, which, although the data were detailed on a wide range of clinical information, might be less accurate and comprehensive than the data obtained from a prospective randomized setting. This may have resulted in residual confounding that could still bias the association direction if there are unmeasured or unknown confounders. Second, information on smoking and drinking habits, body mass index, and clinical biochemical parameters, such as blood glucose, lipids, and uric acid, was unavailable from the NHIRD. All of these are risk factors for CVD and could plausibly also be related with hypnotics use. These confounders cannot be controlled in our multiple analyses, although we have included chronic obstructive pulmonary disease and obesity-related comorbidities as risk factors in the models and adjusted for them in the analyses. Third, patients might be under- or over-diagnosed with different comorbidities, leading to a misclassification bias. Furthermore, for the patients with a combined use of BZDs and non-BZDs, it was unclear whether these medicines were taken simultaneously or in different periods. We were unable to contact the patients directly regarding their use of study drugs because of the anonymity of the identification numbers. In addition, the prescriptions issued before 2004 for the drugs under study were not reflected in our analysis, and this omission could have underestimated the cumulative dosage and may have weakened the observed association. Fourth, we used a propensity score-matching approach to elevate the comparability between the cohorts of hypnotics users and non-users, which potentially led to a multivariable overcontrol that have been regarded as a conservative statistical choice. Each comorbidity does not necessarily precede the use of hypnotics and may be caused by the use of hypnotics, which is likely to raise a question of whether control for comorbidity incidence after hypnotics use may produce overcontrol and thus underestimate the causal associations. Fifth, the relatively small proportion of elderly individuals, who suffer more conventional risk factors, might result in an underestimation of the risk of the incidence of AF. Even so, the effect of hypnotics on the risk of AF was similar and consistent in the young to middle-aged, and even the elderly cohorts (aged 20–39, 40–64, and ≥ 65). Finally, because an increased risk for AF was observed only in the Taiwanese population with the long-term use of hypnotics, the association estimated needs to be cautiously interpreted and further verified in different large-scale cohort populations.

## 5. Conclusions

The prescribed use of hypnotics contributed to the elevated AF risk regardless of gender, age, living religions, clinical comorbidities, and concomitant medications. With unparalleled higher risks of AF, the prescribing of a high-dose use of hypnotics, either or both of BZDs and non-BZDs, should be more cautious in clinical cardiology settings.

## Figures and Tables

**Table 1:** Baseline characteristics of included patients with or without prescription of hypnotics before and after prosperity score matching.

Variables	Before Prosperity Score Matching				After Prosperity Score Matching			
	Total Subjects (*n* = 307,154)	Non-Hypnotics Users (*n* = 219,059)	Hypnotics Users (*n* = 88,095)	*p* ^a^	Total Subjects (*n* = 109,704)	Non-Hypnotics Users (*n* = 54,852)	Hypnotics Users (*n* = 54,852)	*p* ^a^
Demographic parameters, n (%)								
Gender				<0.001				0.88
Female	129,735	87,090 (39.8)	42,645 (48.4)		53,528	26,751 (48.8)	26,777 (48.8)	
Male	177,419	131,969 (60.2)	45,450 (51.6)		56,176	28,101 (51.2)	28,075 (51.2)	
Age, years				<0.001				0.82
20–39	169,360	131,336 (60.0)	38,024 (43.2)		50,087	25,016 (45.6)	25,071 (45.7)	
40–64	118,331	78,724 (35.9)	39,607 (45.0)		47,985	23,989 (43.7)	23,996 (43.7)	
≥65	19,463	8999 (4.1)	10,464 (11.9)		11,632	5847 (10.7)	5785 (10.5)	
Mean (SD)		38.28 (13.42)	44.38 (15.85)			43.67 (15.69)	43.21 (15.50)	
Region				<0.001				0.92
Northern	148,639	108,929 (49.7)	39,710 (45.1)		51,223	25,585 (46.6)	25,638 (46.7)	
Central	58,047	39,407 (18.0)	18,640 (21.2)		21,407	10,702 (19.5)	10,705 (19.5)	
Southern	87,040	61,319 (28.0)	25,721 (29.2)		32,244	16,127 (29.4)	16,117 (29.4)	
Eastern or island	13,428	9404 (4.3)	4024 (4.6)		4830	2438 (4.4)	2392 (4.4)	
Comorbidity, *n* (%)								
Overweight/obesity	1782	1143 (0.5)	639 (0.7)	<0.001	660	328 (0.6)	332 (0.6)	0.87
Diabetics	13,153	6460 (2.9)	6693 (7.6)	<0.001	6521	3299 (6.0)	3222 (5.9)	0.33
Cardiovascular disease	27,491	12,150 (5.5)	15,341 (17.4)	<0.001	13,016	6509 (11.9)	6507 (11.9)	0.99
Chronic obstructive pulmonary disease	12,738	6338 (2.9)	6400 (7.3)	<0.001	5885	2987 (5.4)	2898 (5.3)	0.23
Anxiety	7432	2773 (1.3)	4659 (5.3)	<0.001	2460	1277 (2.3)	1183 (2.2)	0.06
Sleep disorder	87,297	48,834 (22.3)	38,463 (43.7)	<0.001	39,410	19,668 (35.9)	19,742 (36.0)	0.64
Medications, *n* (%)								
Statin	13,713	5604 (2.6)	8109 (9.2)	<0.001	5970	2962 (5.4)	3008 (5.5)	0.54
Glucocorticoid	9882	2714 (1.2)	7168 (8.1)	<0.001	2490	1247 (2.3)	1243 (2.3)	0.94
Nonsteroidal anti-inflammatory drug	63,657	31,911 (14.6)	31,746 (36.0)	<0.001	25,042	12,538 (22.9)	12,504 (22.8)	0.81
Antiplatelet agents	10,652	3375 (1.5)	7277 (8.3)	<0.001	3738	1877 (3.5)	1861 (3.5)	0.79
Anticoagulants	522	116 (0.1)	406 (0.5)	<0.001	132	69 (0.1)	63 (0.1)	0.60
Antidiabetic agents	9144	4166 (1.9)	4978 (5.7)	<0.001	3639	1846 (3.5)	1793 (3.4)	0.37
Anti-Hypertensive drugs	39,551	14,189 (6.5)	25,362 (28.8)	<0.001	15,174	7547 (13.8)	7627 (13.9)	0.48

Antiplatelet agent agents: Clopidogrel/Brilique/Aspirin; Anticoagulants: Warfarin/Rivaroxaban/Pradaxa; Antidiabetic agents: Insulin/oral hypoglycemic drugs; Anti-Hypertensive drugs: Angiotensin-converting enzyme inhibitors/Angiotensin receptor blockers/β-blocker/Calcium entry blockers/Diuretics. ^a^ *p* value was calculated by chi-square test for categorical variables or *t*-test for continuous variables to test the difference between the groups at baseline.

**Table 2:** Stratified analyses of the comparisons with non-hypnotics users for the incident atrial fibrillation in hypnotics users.

	Non-Hypnotics Users			Hypnotics Users			Crude HR (95% CI)	*p*-Value ^a^	Adjusted HR (95% CI)	*p*-Value ^a^	*p*-Value ^b^
	Event	PY	IR	Event	PY	IR					
Overall	166	283,234	5.86	610	319,236	19.11	3.31 (0.79-–3.93)	<0.001	3.61 (3.04–4.28)	<0.001	
Gender											0.65
Female	50	138,048	3.62	203	160,777	12.63	3.48 (2.55–4.75)	<0.001	3.71 (2.72–5.06)	<0.001	
Male	116	145,185	7.99	407	158,459	25.68	3.28 (2.67–4.03)	<0.001	3.56 (2.89–4.37)	<0.001	
Age											0.77
20–39	8	132,597	0.60	23	153,796	1.50	2.56 (1.14–5.73)	0.02	2.66 (1.19–5.95)	0.02	
40–64	57	123,282	4.62	250	137,944	18.12	3.94 (2.95–5.25)	<0.001	3.96 (2.97–5.29)	<0.001	
≥65	101	27,355	36.92	337	27,495	122.57	3.32 (2.66–4.15)	<0.001	3.24 (2.59–4.05)	<0.001	
Region											0.12
Northern	70	133,263	5.25	295	149,375	19.75	3.78 (2.91–4.90)	<0.001	4.08 (3.14–5.30)	<0.001	
Central	36	54,538	6.60	121	62,364	19.40	3.05 (2.10–4.42)	<0.001	3.41 (2.35–4.95)	<0.001	
Southern	50	83,036	6.02	172	93,595	18.38	3.13 (2.28–4.29)	<0.001	3.40 (2.48–4.67)	<0.001	
Eastern or island	10	12,397	8.07	22	13,902	15.83	1.98 (0.94–4.19)	0.07	2.23 (1.05–4.73)	0.04	
Clinical comorbidities											0.09
No	36	159,406	2.26	168	175,210	9.59	4.18 (2.91–5.99)	<0.001	4.29 (2.99–6.16)	<0.001	
Yes	130	123,827	10.50	442	143,026	30.90	3.01 (2.48–3.67)	<0.001	3.14 (2.58–3.82)	<0.001	
Overweight/obesity	3	1630	18.41	5	1628	30.71	1.68 (0.40–7.05)	0.475	3.94 (0.31–9.64)	0.288	
DM	23	15,016	15.32	101	15,844	63.74	4.19 (2.66–6.60)	<0.001	4.14 (2.63–6.53)	<0.001	
CVD	101	29,706	34.00	315	33,045	95.32	2.88 (2.30–3.60)	<0.001	3.02 (2.41–3.78)	<0.001	
COPD	41	13,962	29.37	124	15,052	82.38	2.86 (2.01–4.07)	<0.001	2.95 (2.07–4.21)	<0.001	
Anxiety	5	5757	8.68	16	6467	24.74	3.06 (1.12–8.37)	0.03	3.38 (1.23–9.31)	0.02	
Sleep disorder	12	22,169	5.41	44	33,388	13.18	2.37 (1.25–4.50)	0.01	2.45 (1.28–4.67)	0.01	
Concomitant drugs											0.11
Statin	17	18,618	9.13	37	20,574	17.98	1.88 (1.06–3.34)	0.03	1.97 (1.10–3.52)	0.02	
Glucocorticoid	5	7592	6.59	15	7272	20.63	3.11 (1.13–8.56)	0.03	3.23 (1.17–8.93)	0.02	
Nonsteroid	28	78,805	3.55	79	86,845	9.10	2.50 (1.63–3.85)	<0.001	2.84 (1.84–4.38)	<0.001	
Antiplatelet or anticoagulant agents	19	11,948	15.90	44	11,250	39.11	2.47 (1.44–4.23)	0.001	2.41 (1.39–4.17)	0.002	
Antidiabetic agents	4	12,248	3.27	19	11,132	17.07	5.29 (1.80–15.55)	0.002	6.39 (2.13–19.13)	0.001	
Anti-hypertensive drugs	53	46,228	11.46	169	48,427	34.90	2.99 (2.19–4.07)	<0.001	3.09 (2.26–4.21)	<0.001	

Abbreviations: PY, person-years; IR, incidence rate, per 10,000 person-years; HR, hazard ratio; CI, confidence interval; DM, diabetes mellitus; CVD, cardiovascular disease; COPD, chronic obstructive pulmonary disease. Cardiovascular disease: hypertensive heart disease, ischemic heart disease, pericardium disease, cardiomyopathy, cardiac dysrhythmias, heart failure, complications of heart disease, or cerebrovascular disease; Clinical comorbidity: overweight/obesity, diabetes mellitus, cardiovascular disease, chronic obstructive pulmonary disease, anxiety, or sleep disorder; Antiplatelet agent agents: clopidogrel/brilique/aspirin; Anticoagulants: warfarin/rivaroxaban/pradaxa; Antidiabetic agents: insulin/oral hypoglycemic drugs; Anti-hypertensive drugs: angiotensin-converting enzyme inhibitors/angiotensin receptor blockers/β-blocker/calcium entry blockers/diuretic. ^a^ *p*-value was calculated to indicate a statistical difference for the incident atrial fibrillation between hypnotics users and non-users, using the Cox proportional models without or with multiple adjustments for gender, age, region, clinical comorbidities, and medications at baseline, respectively; ^b^ *p*-value was calculated to indicate a statistical interaction between hypnotics use and each stratum on the incident atrial fibrillation, by using a multivariate-adjusted Cox proportional model with interaction terms.

**Table 3:** Estimated dose–response associations between prescribed use of hypnotics and risk of new-onset atrial fibrillation.

	N	Event	PY	IR	Crude		Adjusted	
					HR (95%CI)	*p* ^a^	HR (95%CI)	*p* ^a^
Non-Hypnotics users	54,852	166	283,234	5.86	1.00 (Ref.)		1.00 (Ref.)	
Isolated BZDs, DDD ^b^								
T1 (<0.22)	11,837	66	86,781	7.61	1.32 (0.99–1.76)	0.05	1.65 (1.24–2.20)	0.001
T2 (0.22–0.74)	12,551	115	70,723	16.26	2.80 (2.20–3.55)	<0.001	3.33 (2.63–4.23)	<0.001
T3 (>0.74)	11,973	264	49,958	52.84	8.84 (7.27–10.73)	<0.001	7.13 (5.86–8.67)	<0.001
Isolated non-BZDs, DDD ^b^								
T1 (<1.66)	766	11	4810	22.87	3.85 (2.09–7.10)	<0.001	7.24 (3.90–13.45)	<0.001
T2 (1.66–4.72)	796	10	4574	21.86	3.69 (1.95–6.99)	<0.001	3.86 (2.03–7.35)	<0.001
T3 (>4.72)	768	14	2287	61.21	10.86 (6.26–18.84)	<0.001	10.68 (6.13–18.62)	<0.001
BZDs and Non-BZDs, DDD ^b^								
T1 (<0.39)	5319	33	38,933	8.48	1.42 (0.97–2.06)	0.07	1.59 (1.09–2.31)	0.02
T2 (0.39–1.65)	5493	45	34,880	12.90	2.17 (1.56–3.02)	<0.001	2.45 (1.75–3.41)	<0.001
T3 (>1.65)	5326	52	26,155	19.88	3.39 (2.48–4.63)	<0.001	3.26 (2.38–4.47)	<0.001

Abbreviations: DDD, defined daily doses; PY, person-years; IR, incidence rate, per 10,000 person-years; HR, hazard ratio; CI, confidence interval; BZDs, benzodiazepines; T, tertiles. ^a^ *p*-value was calculated to indicate a statistical difference for the incident atrial fibrillation in the users across the tertiles of DDD compared with the non-user, using the Cox proportional models without or with multiple adjustments for gender, age, living regions, clinical comorbidities, and concomitant drugs, respectively; ^b^ DDD was classified by the tertiles in the user cohort of isolated BZDs, isolated non-BZDs, and their combination without priorities, respectively.

## Data Availability

The data presented in this study are available on request from the corresponding author. The data are not publicly available due to ethical reasons.

## Supplementary Materials

The following supporting information can be downloaded at: https://www.mdpi.com/article/10.3390/jpm12101645/s1, Figure S1: The flowchart of the present study; Figure S2: Potential biological mechanism through which hypnotics contribute to an increased risk of atrial fibrillation. Hypnotics might be involved in the onset and progression of atrial fibrillation via the hypertensive and (or) arrhythmic effects, which were related to vagal effects and long QT syndrome; Table S1: The hypnotics names with ATC code included in the present study.
