# Supplementary material for: Hypnotics Use Is Associated with Elevated Incident Atrial Fibrillation: A Propensity-Score Matched Analysis of Cohort Study
Source: J Pers Med. 2022 Oct 4;12(10):1645. doi: 10.3390/jpm12101645 (PMC9605069; doi:10.3390/jpm12101645)
Supplement: Supplementary file 1 [file jpm-12-01645-s001.zip › jpm-1807082-supplementary.pdf]

Supplementary Materials: Table S1. The hypnotics names with ATC code included in the present study

Efficacy duration (half-time period)	Drug names	ATC Code
	**Benzodiazepines (BZDs)**	
Long acting (> 24 h)	diazepam	N05BA01
	fludiazepam	N05BA17
	flunitrazepam	N05CD03
	flurazepam	N05CD01
	nitrazepam	N05CD02
	nordazepam	N05BA16
	nimetazepam	N05CD91
	medazepam	N05BA03
Intermediate-acting (8-24 h)	alprazolam	N05BA12
	bromazepam	N05BA08
	estazolam	N05CD04
	lorazepam	N05BA06
	oxazepam	N05BA04
Short-acting (< 8 h)	brotizolam	N05CD09
	midazolam	N05CD08
	triazolam	N05CD05
	**Non-benzodiazepines (Non-BZDs)**	
	zaleplon	N05CF03
	zopiclone	N05CF01
	zolpidem	N05CF02
	eszopiclone	N05CF04

ICD-10-CM, International Classification of Disease, Ninth Revision, Clinical Modification; ATC, Anatomical Therapeutic Chemical Classification

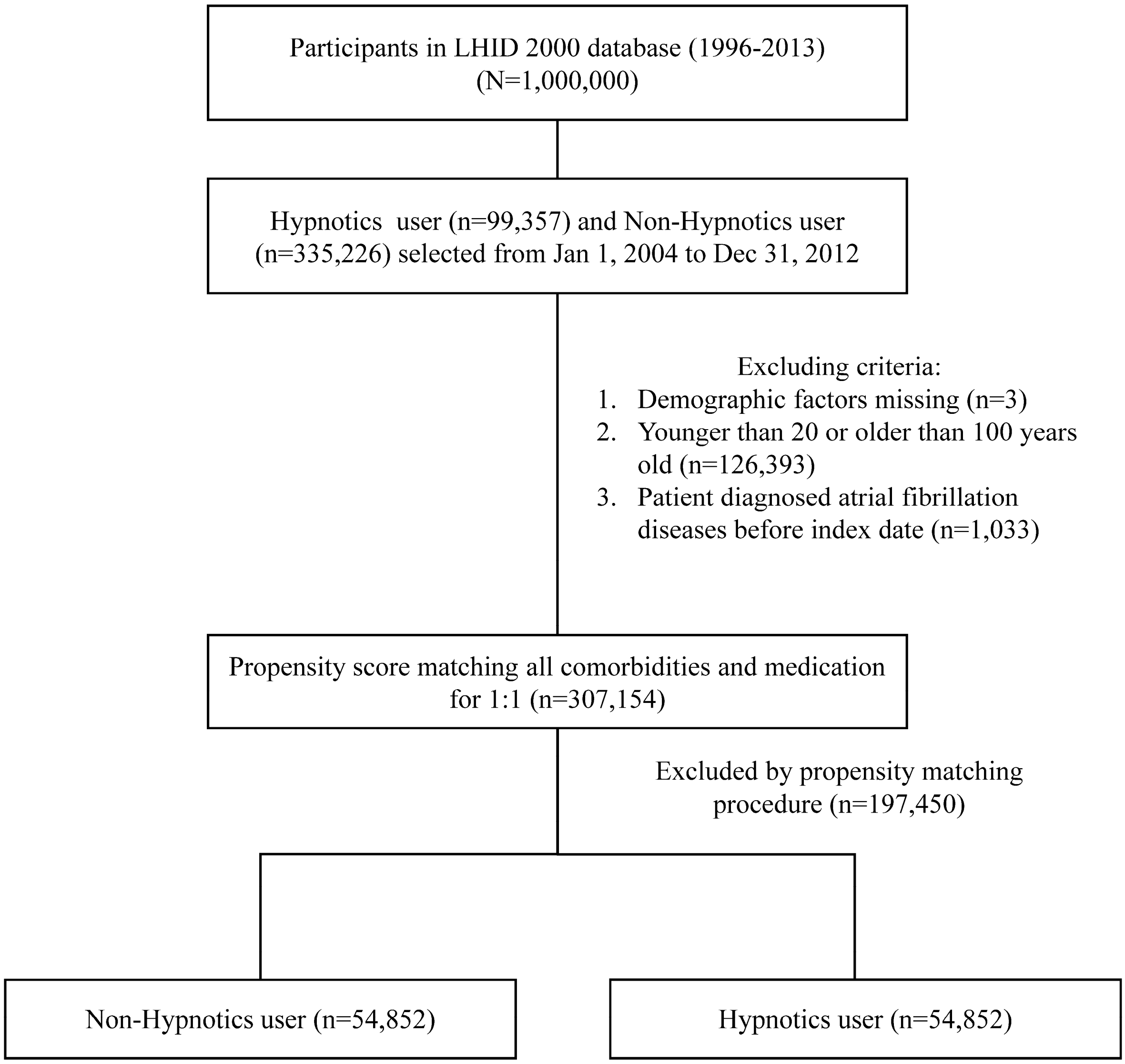

Figure S1. The flowchart of the present study.

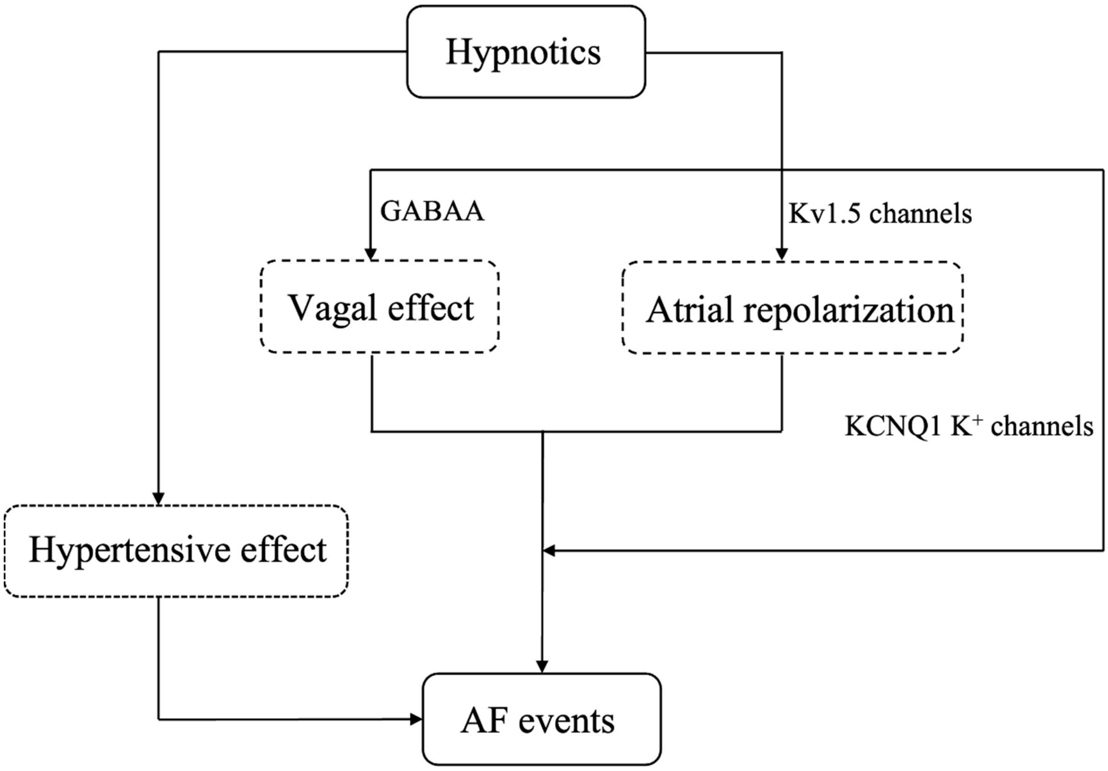

Figure S2. Potential biological mechanism through which hypnotics contribute to an increased risk of atrial fibrillation. Hypnotics might be involved in the onset and progression of atrial fibrillation via the hypertensive and (or) arrhythmic effects, which were related to vagal effects and long QT syndrome.
